# Origin Traceability of Chinese Mitten Crab (*Eriocheir sinensis*) Using Multi-Stable Isotopes and Explainable Machine Learning

**DOI:** 10.3390/foods14142458

**Published:** 2025-07-13

**Authors:** Danhe Wang, Chunxia Yao, Yangyang Lu, Di Huang, Yameng Li, Xugan Wu, Weiguo Song, Qinxiong Rao

**Affiliations:** 1 The Institute of Agro-Food Standards and Testing Technology, Shanghai Academy of Agricultural Sciences, Shanghai 201403, China; wdh@saas.sh.cn (D.W.); chunxiayao2007@saas.sh.cn (C.Y.); yyanglz@163.com (Y.L.); hdsaas@126.com (D.H.); liyameng@saas.sh.cn (Y.L.); 2 Shanghai Service Platform of Agro-Products Quality and Safety Evaluation Technology, Shanghai 201403, China; 3 Research Centre on Fish Nutrition and Environmental Ecology of Ministry of Agriculture and Rural Affairs, Shanghai Ocean University, Shanghai 201306, China; xgwu@shou.edu.cn; 4 Shanghai Co-Elite Agri-Food Testing Technical Service Co., Ltd., Shanghai 201403, China

**Keywords:** Chinese mitten crab, origin traceability, stable isotopes, explainable machine learning, tissue specificity

## Abstract

The Chinese mitten crab (*Eriocheir sinensis*) industry is currently facing the challenges of origin fraud, as well as a lack of precision and interpretability of existing traceability methods. Here, we propose a high-precision origin traceability method based on a combination of stable isotope analysis and interpretable machine learning. We sampled Chinese mitten crabs from six origins representing diverse aquatic environments and farming practices, and analyzed their *δ*^13^C, *δ*^15^N, *δ*^2^H, and *δ*^18^O stable isotope compositions in different sexes and tissues (hepatopancreas, muscle, and gonad). By comparing the classification performance of Random Forest, XGBoost, and Logistic Regression models, we found that the Random Forest model outperformed the others, achieving high accuracy (91.3%) in distinguishing samples from different origins. Interpretation of the optimal Random Forest model, using SHAP (SHapley Additive exPlanations) analysis, identified *δ*^2^H in male muscle, *δ*^15^N in female hepatopancreas, and *δ*^13^C in female hepatopancreas as the most influential features for discriminating geographic origin. This analysis highlighted the crucial role of environmental factors, such as water source, diet, and trophic level, in origin discrimination and demonstrated that isotopic characteristics of different tissues provide unique discriminatory information. This study offers a novel paradigm for stable isotope traceability based on explainable machine learning, significantly enhancing the identification capability and reliability of Chinese mitten crab origin traceability, and holds significant implications for food safety assurance.

## 1. Introduction

Accurate verification of the geographical origin of aquatic products is crucial for establishing robust food traceability systems, tracing potential pollutant sources, protecting ecological environments, and upholding consumer rights to information [1,2,3]. Reliable origin information underpins various certification and labeling programs (e.g., organic, sustainable certifications), contributing to the promotion of ethical and sustainable aquaculture practices, and is directly linked to maintaining market order and fostering consumer trust [4,5]. The Chinese mitten crab (*Eriocheir sinensis*), recognized for its broad adaptability and substantial economic value, holds a significant place in global aquaculture, and is particularly popular in China and East Asia [6,7,8,9]. However, the Chinese mitten crab market is significantly impacted by origin fraud and mislabeling [10,11,12]. This widespread practice involves crabs from less reputed or lower-cost farming areas being falsely marketed as premium products from famous regions, such as Yangcheng Lake, severely undermining the credibility of genuine regional brands and disrupting fair market competition [11,12,13]. Beyond economic concerns, Chinese mitten crabs inhabit diverse environments, including areas potentially subject to pollution from industrial or agricultural activities. As these crabs can accumulate contaminants, such as heavy metals, and persistent organic pollutants, including dioxins, those sourced from polluted areas pose a significant health risk if they enter the food supply chain without proper identification and safety checks [14,15]. Therefore, ensuring the accurate geographical origin identification and robust traceability of Chinese mitten crabs is not merely a matter of consumer rights and fair trade, but an imperative step for safeguarding public health against potentially contaminated products.

Chinese mitten crabs’ variability in farming environments presents a challenge for accurate geographical origin identification. Currently, Chinese mitten crabs available in the market predominantly come from high-yield, easily managed pond farming settings, resulting in prior origin tracing studies largely concentrating on such samples [12,16,17,18]. Lake enclosure farming (like the renowned Yangcheng Lake model) acts as a supplement, often involving more extensive management techniques [19]. Conversely, natural river populations are limited in large-scale cultivation due to their migratory behaviors and the difficulty in controlling environmental factors. Furthermore, rice field farming, an environmentally friendly method, is gradually increasing its market share [20]. The complexity of these farming origins suggests that models for origin discrimination built solely on a single farming type (especially ponds) may have limited generalizability and may not accurately represent actual market conditions.

Stable Isotope Analysis (SIA), a potent geochemical tracing technique, has been widely employed in validating food authenticity, particularly for determining geographical origin [18,21,22,23]. The principle is that the isotopic composition of biological tissues (e.g., carbon *δ*^13^C, nitrogen *δ*^15^N, hydrogen *δ*^2^H, oxygen *δ*^18^O, and sulfur *δ*^34^S) reflects the geochemical characteristics of their growth environment (climate, geology, and water sources), as well as their food sources and trophic levels [24]. Regional variations in these factors, combined with specific farming practices (e.g., feed composition and water management), create distinct ‘isotopic fingerprints’ capable of differentiating aquatic products from various geographical origins or production systems. Previous research has attempted to use *δ*^13^C and *δ*^15^N ratios in Chinese mitten crab muscle tissue (typically walking leg muscle) to determine origin, successfully distinguishing some crabs from different farming locations [12,18]. These distinctions were primarily linked to differences in feed sources or specific aquaculture management approaches [25]. However, while hydrogen (*δ*^2^H) and oxygen (*δ*^18^O) isotopes can indicate hydrological cycle features, their ratios can fluctuate due to short-term precipitation events and water source mixing, limiting their consistent application in complex scenarios, such as differentiating farming environments [26]. Consequently, most models have predominantly relied on *δ*^13^C and *δ*^15^N [18,27]. Despite the significant potential of SIA for origin determination, existing studies on Chinese mitten crab have clear limitations. Firstly, current models are often developed using easily obtained pond-farmed samples, meaning their discriminatory power might reflect the common traits of controlled aquaculture settings (such as isotopic signals dominated by artificial feed) rather than genuine geographical origin signals [12,17,18,28]. Secondly, Chinese mitten crabs sold in the market can originate from natural or semi-natural bodies of water, such as lakes and rivers [12,18,28]. The biogeochemical processes and food web structures in these environments differ considerably from intensively managed pond systems, and their isotopic signatures may not be adequately represented in existing models, leading to reduced accuracy when applied to non-pond samples. Furthermore, prior studies commonly analyzed only a single tissue (e.g., muscle), overlooking the isotopic variations among different tissues (such as hepatopancreas and gonads), which arise from differing metabolic rates and nutrient integration times [12,18,28]. This tissue-specific isotopic variability could contain origin information but has not been fully utilized, thus limiting the precision and robustness of discrimination models.

To address the aforementioned limitations and improve the accuracy and generalizability of geographical origin discrimination models for Chinese mitten crab, this study aims to increase the diversity of sample sources by including samples from various typical habitats, specifically ponds, natural lakes, and the main stem of the Yangtze River. Furthermore, given that the tissue-specific isotopic turnover rates in Chinese mitten crabs are not yet well-characterized, it remains unclear which tissue most effectively records long-term geographical signals versus recent environmental shifts. Since the isotopic signatures of edible tissues are directly pertinent to food authenticity verification, understanding the distinct isotopic behaviors of these tissues is critical. Therefore, this study analyzed the *δ*^13^C, *δ*^15^N, *δ*^2^H, and *δ*^18^O stable isotope ratios in three edible tissues (muscle, hepatopancreas, and gonad) from both female and male crabs to reveal multi-tissue, multi-sex isotopic fractionation patterns and their potential for origin traceability. Moving beyond traditional multivariate statistical methods, this research employed explainable machine learning (XML) techniques [29]. XML offers advantages in handling high-dimensional, non-linear isotopic data and uncovering intricate patterns. It can also explicitly determine the contribution of each individual feature (e.g., specific isotope in a particular tissue and sex) to the classification of origin. This allows for the identification of the primary features most crucial for origin discrimination, while also facilitating the optimization of model performance through feature engineering and the exploration of effective feature combinations. The primary objectives of this study were (1) to enhance the accuracy and cross-environment applicability of Chinese mitten crab geographical origin discrimination; (2) to mitigate the deficiencies of existing models in distinguishing samples from natural water bodies; and (3) to provide more reliable scientific evidence for the sustainable management, brand protection, and product quality control of the Chinese mitten crab industry.

## 2. Materials and Methods

### 2.1. Experimental Materials

**Figure 1:**
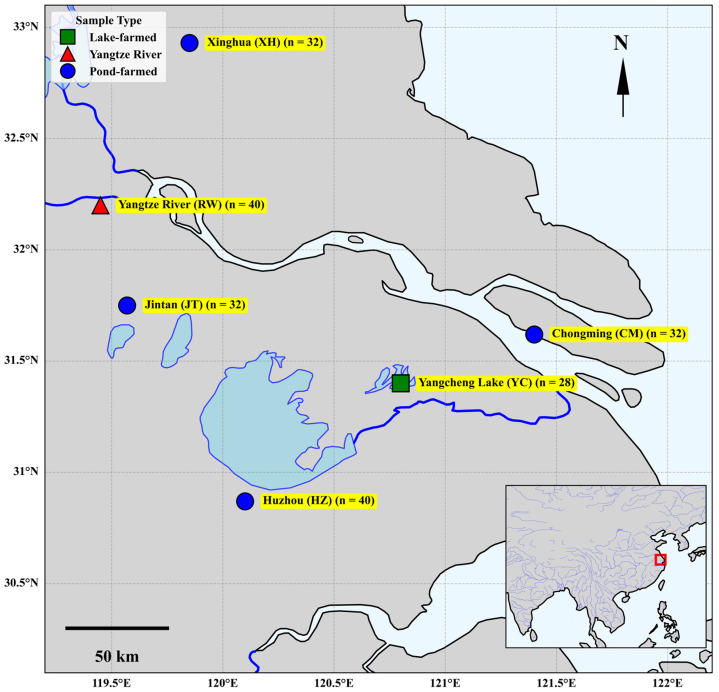
Map of the geographical locations and sample types of Chinese mitten crabs collected from six origins in the lower Yangtze River basin. Blue circles represent pond-farmed origins: Jintan (JT, n = 32), Huzhou (HZ, n = 40), Chongming (CM, n = 32), and Xinghua (XH, n = 32). A green square indicates the lake-farmed origin: Yangcheng Lake (YC, n = 28). A red triangle denotes the wild Yangtze River origin: Yangtze River (RW, n = 40).

Samples of Chinese mitten crab (*Eriocheir sinensis*) were collected from major pond aquaculture areas and natural habitats in the lower Yangtze River Basin (Figure 1). Due to the potential for sample mortality during transport, the final sample size was determined based on the number of viable individuals upon arrival. Pond-farmed individuals were sampled in November 2021 from Jintan (JT; 16 females, 16 males), Huzhou (HZ; southern Taihu Lake region; 20 females, 20 males), Chongming (CM; 16 females, 16 males), and Xinghua (XH; 16 females, 16 males), with sexually mature individuals of both sexes collected at each site. Lake-farmed samples were obtained from Yangcheng Lake (YC; Suzhou, China; 14 females, 14 males) in November 2021. Wild individuals were captured from the Yangtze River (RW; 20 females, 20 males) in the Zhenjiang section of the river in November 2019. All samples were transported alive to the laboratory. Upon arrival, surface water was wiped off, and individuals were anesthetized under cold conditions prior to dissection. Hepatopancreas (H), gonad (G), and muscle (M) tissues were excised, lyophilized at −40 °C, ground to pass through a <100-mesh sieve, and stored in a constant-temperature drying oven until stable isotope analysis.

### 2.2. Isotope Analysis

#### 2.2.1. *δ*^13^C and *δ*^15^N Analysis

For the analysis of *δ*^13^C and *δ*^15^N isotope ratios, all samples underwent lipid extraction following the protocol recommended by [30] to correct *δ*^13^C values and minimize shifts in *δ*^15^N. Chloroform–methanol extraction was conducted based on the original method of [31]. Each sample (0.2 mg) was weighed into a tin capsule and introduced into an elemental analyzer (Flash IRMS, Thermo Fisher Scientific, Bremen, Germany) via an autosampler, where it was combusted and reduced to CO_2_ and N_2_ gases. These gases were then analyzed using a stable isotope ratio mass spectrometer (Delta V Advantage, Thermo Fisher Scientific, Bremen, Germany). The combustion furnace was maintained at 980 °C and the column oven at 60 °C. Helium (He) was used as the carrier gas at a flow rate of 180 mL/min. Reference CO_2_ and N_2_ gases were introduced at flow rates of 50 mL/min. Stable isotope values were calibrated using a multi-point normalization approach with international reference materials from the United States Geological Survey: USGS40 (L-glutamic acid, *δ*^13^C = −26.38‰, *δ*^15^N = −4.5‰), USGS90 (millet flour, *δ*^13^C = −13.75‰, *δ*^15^N = +8.84‰), and USGS91 (rice flour, *δ*^13^C = −28.28‰, *δ*^15^N = +1.78‰). USGS40 was additionally used as a quality control standard. Blanks and internal standards were analyzed concurrently for result correction. Analytical precision was better than ±0.15‰ for both *δ*^13^C and *δ*^15^N.

#### 2.2.2. *δ*^2^H and *δ*^18^O Analysis

For the analysis of *δ*^2^H and *δ*^18^O, 0.2 mg of each sample was weighed into a silver capsule. The samples were introduced into the isotope analyzer via an autosampler and pyrolyzed in a high-temperature elemental analyzer (Flash IRMS, Thermo Fisher Scientific, Bremen, Germany) to produce H_2_ and CO gases. These gases were then transferred to a stable isotope ratio mass spectrometer (Delta V Advantage, Thermo Fisher Scientific, Bremen, Germany) for detection. The pyrolysis furnace was maintained at 1350 °C, and the column oven at 80 °C. Helium served as the carrier gas at a flow rate of 100 mL/min.

Isotope values were calibrated using a multi-point normalization method with international reference materials from the United States Geological Survey (USGS), including USGS55 (wood powder, *δ*^2^H = −28.2‰, *δ*^18^O = +19.12‰), USGS90 (millet flour, *δ*^2^H = −13.9‰, *δ*^18^O = +35.9‰), and USGS91 (rice flour, *δ*^2^H = −45.7‰, *δ*^18^O = +21.13‰). USGS90 was used for quality control. Procedural blanks and internal standards were applied to correct for background signals and instrumental drift. The analytical precision was better than ±3‰ for *δ*^2^H and ±0.4‰ for *δ*^18^O.

Stable isotope values are reported in delta (*δ*) notation, representing the relative difference between the isotope ratio of the sample and that of an international standard, calculated as:

(1) $$\delta ‰=\left(\frac{R_{sample}}{R_{standard}}-1\right)$$

where *R_sample_* and *R_standard_* refer to the ratio of heavy to light isotopes in the sample and standard, respectively (i.e., ^2^H/^1^H and ^18^O/^16^O). To improve measurement accuracy and minimize matrix effects, *δ*^2^H and *δ*^18^O values, as well as *δ*^13^C and *δ*^15^N values, were all determined using a three-point calibration approach.

#### 2.2.3. Assumption Testing and Comparative Statistical Analysis

To establish a foundational understanding of isotopic variability and identify potential discriminatory variables for subsequent machine learning model construction, initial comparative statistical analyses were performed. These analyses aimed to quantify significant differences in stable isotope ratios (*δ*^13^C, *δ*^15^N, *δ*^2^H, and *δ*^18^O) attributable to geographical origin, aquatic environmental type, and tissue specificity. Identifying such statistically significant variations is crucial as it provides the basis for effective feature engineering and validates the selection of these parameters for origin traceability.

Prior to conducting inter-group difference analyses, the data were subjected to statistical assumption testing, including assessments for normality and homogeneity of variances. For each sample group, the Shapiro–Wilk test was employed to evaluate whether the variable distributions conformed to a normal distribution. A *p*-value greater than 0.05 was interpreted as indicating no significant deviation from normality. Homogeneity of variances across groups was assessed using Levene’s test. If the *p*-value exceeded 0.05, the variances among groups were considered not significantly different, thereby satisfying the assumption of homoscedasticity. Based on the outcomes of the normality and homogeneity of variance tests, the following strategy was adopted for inter-group difference analysis. If both normality and homogeneity of variance assumptions were met, one-way analysis of variance (ANOVA) was utilized to test for significant differences among groups. If either assumption was not satisfied, the non-parametric Kruskal–Wallis test was employed. In instances where the Kruskal–Wallis test indicated significant differences, Dunn’s post-hoc multiple comparison test, with Bonferroni correction for familywise error rate, was subsequently performed for pairwise inter-group comparisons. Based on the prerequisite analyses, the Kruskal–Wallis test was used for difference analysis in this study. The results of assumption checks and the corresponding statistical tests for each comparison are summarized in Supplementary Table S1.

All statistical analyses were conducted within a Python 3.12 environment, utilizing open-source statistical libraries including scipy, statsmodels, scikit-posthocs, and statannotations. The significance level (α) was set at 0.05.

### 2.3. Chinese Mitten Crab Origin Classification Models

#### 2.3.1. Machine Learning Approach

Tree-based ensemble models have previously demonstrated strong classification performance in multi-origin traceability tasks for aquatic products [32,33,34]. Therefore, this study selected two representative tree-based models, Random Forest (RF, from scikit-learn version 1.5.1) and Extreme Gradient Boosting (XGBoost, version 2.1.2), as the primary classification algorithms, utilizing their non-linear modeling capabilities and ability to capture complex feature interactions for the Chinese mitten crab origin classification task. Additionally, considering that the features in this study involved stable isotope measurements from different body tissues (hepatopancreas, muscle, and gonad), and these tissues may respond differently to environmental signals during physiological functions and metabolic processes, potentially introducing feature combinations with tissue-specific or inherent linear relationships, Logistic Regression (LR, from scikit-learn version 1.5.1), a linear model, was included in this evaluation to investigate whether this data structure could be effectively captured by a simpler linear model and to provide a baseline for model selection.

Random Forest (RF) is a classical ensemble learning algorithm that constructs numerous decision trees and determines the final prediction result through majority voting (in classification tasks). RF is robust, less prone to overfitting, and effective in handling high-dimensional data and non-linear relationships between features, with built-in feature importance assessment capabilities [35].

Extreme Gradient Boosting (XGBoost) is an efficient and powerful ensemble learning algorithm based on the gradient boosting framework. It iteratively builds decision trees, optimizing the objective function at each step while incorporating regularization techniques to prevent overfitting. XGBoost demonstrates excellent performance in handling structured data [36].

Logistic Regression establishes a linear relationship between features and target class probabilities using a logistic function for classification. It directly measures the strength of linear associations between features and classes, aiding in understanding whether simple separable linear patterns exist in the data [37].

In this study, the dataset was randomly split into training and test sets at a 7:3 ratio. To address potential sample imbalance across origins (e.g., due to transportation-related loss), stratified K-fold cross-validation was employed during model training and evaluation to preserve the class distribution across all folds. Hyperparameter tuning for each model was conducted using grid search combined with stratified K-fold cross-validation on the training data, aiming to optimize predictive performance while controlling for overfitting and enhancing generalization. Details on hyperparameter settings and data preprocessing are provided in Supplementary Table S2.

#### 2.3.2. Classification Features

**Figure 2:**
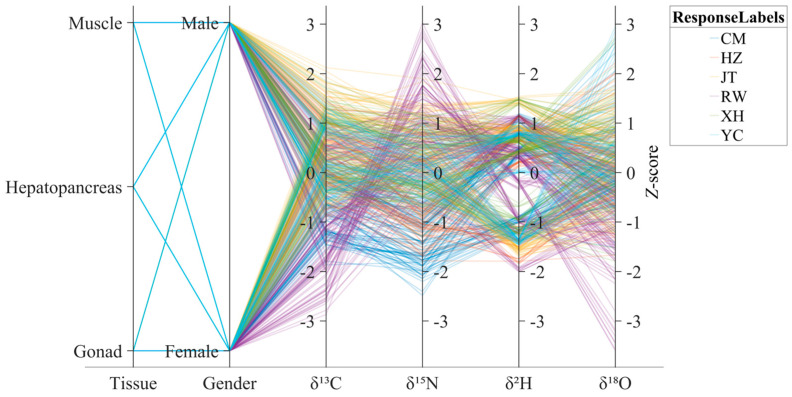
Parallel coordinate plot illustrating the multi-dimensional feature space and the distribution of samples from different origins. Each line represents a sample, colored according to its origin (see legend for color mapping to origin labels), connecting its values across the axes representing Tissue type (Muscle, Hepatopancreas, or Gonad), Sex (Male or Female), *δ*^13^C, *δ*^15^N, *δ*^2^H, and *δ*^18^O values.

The *δ*^13^C, *δ*^15^N, *δ*^2^H, and *δ*^18^O stable isotope values measured from different sexes (female and male) and body tissues (muscle, gonad, and hepatopancreas) of Chinese mitten crab samples were used as classification features. These measurements were systematically combined according to sex and tissue type to construct distinct classification variables. This strategy yielded 24 unique features, each representing the isotopic composition of a specific isotope within a defined tissue and sex group. The structure of these features is illustrated in Figure 2 via a parallel coordinate plot, which visualizes the distribution patterns of samples from different origins across the multi-dimensional feature space. This feature engineering approach was designed to comprehensively evaluate the discriminatory power of isotopic information embedded in different tissues and sexes for geographic origin classification. The rationale was that variations in metabolic rate and elemental turnover across tissues may result in distinct capacities to record environmental isotopic signals [38,39]. Each feature thus represented a unique isotopic fingerprint shaped by both biological and environmental influences. These differentiated feature combinations provided multi-dimensional, tissue- and sex-specific isotopic evidence, offering informative inputs for origin discrimination modeling.

#### 2.3.3. Model Evaluation

In this study, we employed four classification evaluation metrics to quantify the performance of different models. These metrics were primarily derived from the Confusion Matrix. The core evaluation metrics included: Area Under the Receiver Operating Characteristic Curve (AUC), Accuracy, Recall (also known as Sensitivity), and F1 Score.

Accuracy quantified the proportion of samples correctly classified by the model across all classes.

(2) $$\mathrm{Accuracy}=\frac{TP}{TS}$$

where *TP* represented the total number of samples correctly assigned to their true classes, and *TS* was the total number of samples evaluated.

Recall (or Sensitivity), also calculated per class, measured the proportion of actual samples belonging to a specific class that were correctly identified by the model.

(3) $$\mathrm{Recall}=\frac{TP}{TP+FN}$$

where *TP* was the number of true positives and *FN* was the number of false negatives for that class.

The F1 Score, the harmonic mean of precision and recall, provided a comprehensive evaluation metric per class, balancing both precision and recall.

(4) $$F1=\frac{2\times Precision\times Recall}{\mathrm{Precision}+\mathrm{Recall}}$$

The F1 Score is particularly suitable for evaluating performance in situations where sample counts across different classes may be imbalanced, offering a thorough quantitative assessment of the model’s classification accuracy and completeness. These evaluation metrics collectively allowed us to quantify the models’ performance in the multi-origin classification task and analyze their capabilities in terms of correct classification and error types, thereby identifying the optimal model for further analysis.

#### 2.3.4. Model Interpretation Using SHAP

Based on the model performance after hyperparameter optimization, the best-performing model (Random Forest) was selected for further interpretation to understand its decision-making process. We employed the SHapley Additive exPlanations (SHAP) method, a state-of-the-art approach for explaining the output of machine learning models. SHAP improves upon previous local explanation methods by providing a unified framework for interpreting model predictions. The contribution of each feature (variable) to the model’s output was evaluated using the SHAP algorithm, which is based on Shapley values from cooperative game theory [40]. For tree-based models like Random Forest, efficient algorithms such as TreeExplainer are available to compute exact SHAP values. In this study, SHAP values were computed for each prediction to explain how individual features contributed to classifying samples into different origins.

## 3. Results and Discussion

### 3.1. Stable Isotope Values of Chinese Mitten Crabs from Different Origins

**Figure 3:**
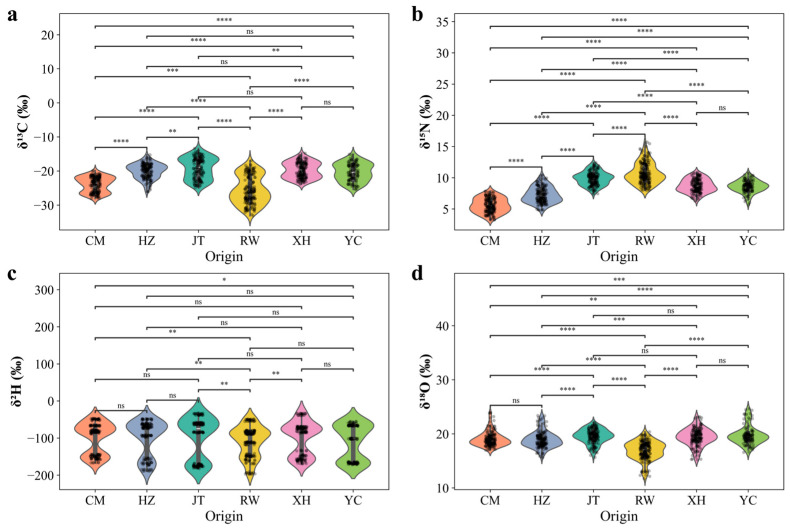
Stable isotope composition of Chinese mitten crabs from different origins. (**a**–**d**) Violin plots with overlaid data points illustrating the stable isotope values for (**a**) *δ*^13^C, (**b**) *δ*^15^N, (**c**) *δ*^2^H, and (**d**) *δ*^18^O across six origins (CM, HZ, JT, RW, XH, YC). Statistical significance of differences between origins is indicated by connecting lines and asterisks (* *p* < 0.05, ** *p* < 0.01, *** *p* < 0.001, **** *p* < 0.0001), while ‘ns’ denotes no significant difference.

The stable isotope compositions (*δ*^13^C, *δ*^15^N, *δ*^2^H, and *δ*^18^O) of Chinese mitten crab samples from different geographic origins are summarized in Figure 3. The mean (± SD) values for each origin were as follows: CM, −24.00 ± 2.25‰, 5.58 ± 1.13‰, −99.91 ± 41.31‰, and 19.06 ± 1.33‰; HZ, −20.18 ± 2.17‰, 7.15 ± 1.41‰, −99.87 ± 47.61‰, and 18.83 ± 1.57‰; JT, −18.96 ± 3.19‰, 9.93 ± 1.04‰, −99.02 ± 55.76‰, and 19.50 ± 1.33‰; RW, −25.56 ± 4.11‰, 10.72 ± 1.70‰, −109.53 ± 41.09‰, and 16.93 ± 1.74‰; XH, −19.67 ± 2.38‰, 8.74 ± 1.08‰, −100.54 ± 44.68‰, and 19.40 ± 1.62‰; and YC, −20.39 ± 2.68‰, 8.56 ± 1.04‰, −110.64 ± 47.57‰, and 19.70 ± 1.71‰ for *δ*^13^C, *δ*^15^N, *δ*^2^H, and *δ*^18^O, respectively.

Statistical analysis (Figure 3a–d) revealed significant variation in *δ*^13^C values among origins (*p* < 0.001), with the exception of no significant differences among HZ, YC, and XH (*p* > 0.05). The *δ*^13^C values of RW samples were significantly lower than those of all other origins. In contrast, the higher *δ*^13^C values observed in samples from CM, HZ, JT, XH, and YC may reflect greater reliance on artificial feed or primary producers with elevated *δ*^13^C values in these farming systems [41,42]. No statistically significant difference between YC and XH (0.01 < *p* < 0.05) may suggest similar food sources (Figure 3a).

Analysis of *δ*^15^N values also revealed significant differences among origins (*p* < 0.001; Figure 3b). The lowest *δ*^15^N values were observed in samples from CM, while the highest were recorded in those from the RW. These differences likely reflect variations in food web trophic structures or baseline nitrogen sources across regions [43]. In particular, the elevated *δ*^15^N values in wild RW crabs may suggest a higher trophic position or distinct nitrogen inputs compared to farmed populations. No significant differences in *δ*^15^N were found between YC and XH (*p* > 0.05). When considered alongside the relatively small *δ*^13^C differences between these two origins (*p* > 0.05), the data suggest that crabs from YC and XH may share similar food sources or occupy comparable trophic positions.

*δ*^2^H and *δ*^18^O stable isotope compositions primarily reflect the isotopic characteristics of the environmental water body, with differences related to water source recharge, evaporation rate, and other hydrogeochemical processes [26]. The *δ*^2^H values primarily reflect the isotopic characteristics of environmental water bodies, influenced by factors such as recharge sources, evaporation rates, and hydrogeochemical processes. As shown in Figure 3c, significant differences in *δ*^2^H were observed between samples from CM, YC, and the RW (*p* < 0.05), while no significant differences were found among other origins. Chongming’s unique estuarine environment at the mouth of the Yangtze River, characterized by brackish water and a dynamic hydrological regime, may account for the observed *δ*^2^H differences relative to inland freshwater sites. In addition, RW samples exhibited significant *δ*^2^H differences compared to those from JT, XH, CM, and HZ (*p* < 0.05). In contrast, several other pairwise comparisons, such as between CM and YC or HZ and JT, did not show significant differences (Figure 3c). This highlights the sensitivity of *δ*^2^H signatures to hydrological context, even among geographically proximate locations. The *δ*^18^O values similarly reflect environmental water inputs and hydroclimatic processes. As shown in Figure 3d, significant differences in *δ*^18^O values were detected among most origins (*p* < 0.05), except between CM and HZ, JT and YC, and JT and XH (*p* > 0.05). Notably, wild samples from the RW exhibited the lowest mean *δ*^18^O (16.93 ± 1.74‰) and *δ*^2^H (−109.53 ± 41.09‰) values among all groups. These lower values may reflect the distinct hydrological recharge and reduced evaporation intensity in the Yangtze River mainstream [44].

### 3.2. Stable Isotope Differences of Chinese Mitten Crabs Across Water Body Types

**Figure 4:**
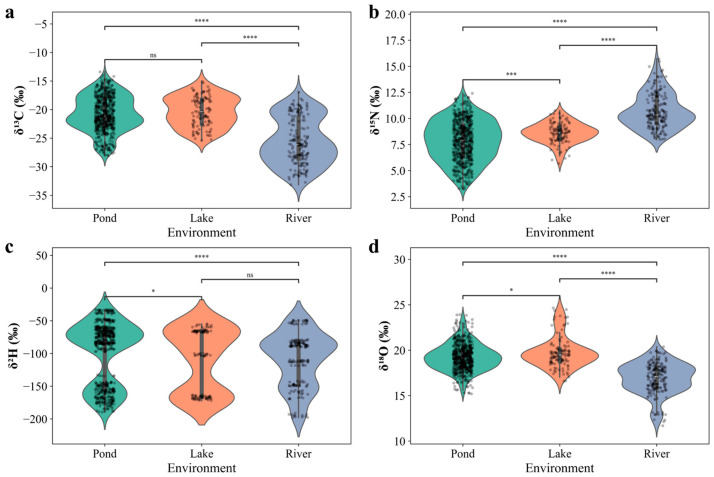
Stable isotope composition of Chinese mitten crabs across different water body types. (**a**–**d**) Violin plots with overlaid data points illustrating the stable isotope values for (**a**) *δ*^13^C, (**b**) *δ*^15^N, (**c**) *δ*^2^H, and (**d**) *δ*^18^O across three environmental types (1, 2, 3), representing different farming modes or habitats. Statistical significance of differences between environments is indicated by connecting lines and asterisks (* *p* < 0.05, *** *p* < 0.001, **** *p* < 0.0001), while ‘ns’ denotes no significant difference.

A comparative analysis of stable isotope characteristics of samples from typical water body types (pond farming, lake farming, and wild Yangtze River) was further conducted (Figure 4). Statistical results indicated significant differences in the stable isotope composition of Chinese mitten crabs across these different aquatic environments. Specifically, the mean (± standard deviation) values for *δ*^13^C, *δ*^15^N, *δ*^2^H, and *δ*^18^O of pond farming samples were −20.74 ± 3.19‰, 7.83 ± 2.02‰, −99.84 ± 47.46‰, and 19.20 ± 1.49‰, respectively. Lake farming samples showed corresponding values of −20.39 ± 2.63‰, 8.56 ± 1.04‰, −110.64 ± 47.57‰, and 19.70 ± 1.71‰. Wild samples from the Yangtze River exhibited distinct isotopic characteristics: *δ*^13^C of −25.56 ± 4.10‰, *δ*^15^N of 10.72 ± 1.70‰, *δ*^2^H of −109.53 ± 41.09‰, and *δ*^18^O of 16.93 ± 1.74‰. The Kruskal–Wallis test (Figure 4a,b,d) revealed statistically significant differences in the *δ*^13^C, *δ*^15^N, and *δ*^18^O isotope values among Chinese mitten crabs from these three main environmental types (*p* < 0.05).

Chinese mitten crabs from the Yangtze River environment exhibited the lowest *δ*^13^C values (mean −25.56‰) (Figure 4a). This may suggest that their food sources relied more heavily on primary producers or organic matter input with lower *δ*^13^C values, such as C_3_ plant detritus or certain types of phytoplankton common in large riverine ecosystems. Large river basins often receive substantial terrestrial organic matter input, which typically has lower *δ*^13^C characteristics, potentially directly or indirectly influencing the baseline isotopic signals of the food web [45]. In contrast, crabs from pond and lake farming environments showed relatively higher *δ*^13^C values [41,42]. This was likely related to the extensive use of artificial feed in these farming modes, which often contain components from C_4_ plants (with typical *δ*^13^C values between −10‰ and −16‰) or mixed sources, leading to the relative enrichment of ^13^C in the consumer tissues.

Regarding *δ*^15^N (Figure 4b), Yangtze River samples showed the highest mean value (10.72‰), significantly exceeding those from ponds (7.83‰) and lakes (8.56‰). Higher *δ*^15^N values are generally associated with consumers occupying higher trophic levels in the food web. However, if interpreted solely from a trophic perspective, the relatively low *δ*^13^C values of Yangtze River samples appear somewhat contradictory to their high *δ*^15^N values (as increasing trophic level typically involves an enrichment of approximately 3–5‰ in *δ*^15^N and 0–1‰ in *δ*^13^C). This suggested that the nitrogen sources in the Yangtze River water might be more complex. As an open large river system, the Yangtze River basin receives substantial input of nitrogen with higher *δ*^15^N values from extensive anthropogenic activities (e.g., domestic sewage and agricultural runoff), which can elevate the baseline *δ*^15^N values of the water body, subsequently affecting the *δ*^15^N composition of crabs residing within it, even if they do not occupy the highest trophic levels [46,47]. In contrast, *δ*^15^N values in relatively closed or semi-closed water bodies like ponds and lakes may be lower due to controlled anthropogenic inputs or natural background sources.

The *δ*^2^H and *δ*^18^O values, reflecting the isotopic composition of environmental water bodies, also differed across water body types. Samples from the Yangtze River had lower *δ*^2^H (−109.53‰) and *δ*^18^O (16.93‰) values than those from ponds and lakes (Figure 4c,d). Water body *δ*^2^H and *δ*^18^O values are primarily influenced by local precipitation characteristics, evaporation rate, runoff mixing, and water exchange rates. Yangtze River water may be more influenced by its upstream sources (e.g., meltwater from high mountains or inland precipitation, which typically have lower *δ*^2^H and *δ*^18^O values). Furthermore, the rapid water turnover rate in large rivers can limit the enrichment of heavy isotopes (^18^O and ^2^H) due to evaporation [44]. In contrast, relatively stagnant or semi-closed water bodies such as ponds and lakes experience stronger evaporation, leading to the enrichment of heavy isotopes in the remaining water and consequently in the tissues of crabs living within them, resulting in higher *δ*^2^H and *δ*^18^O values.

### 3.3. Stable Isotope Differences Across Tissues in Chinese Mitten Crabs

**Figure 5:**
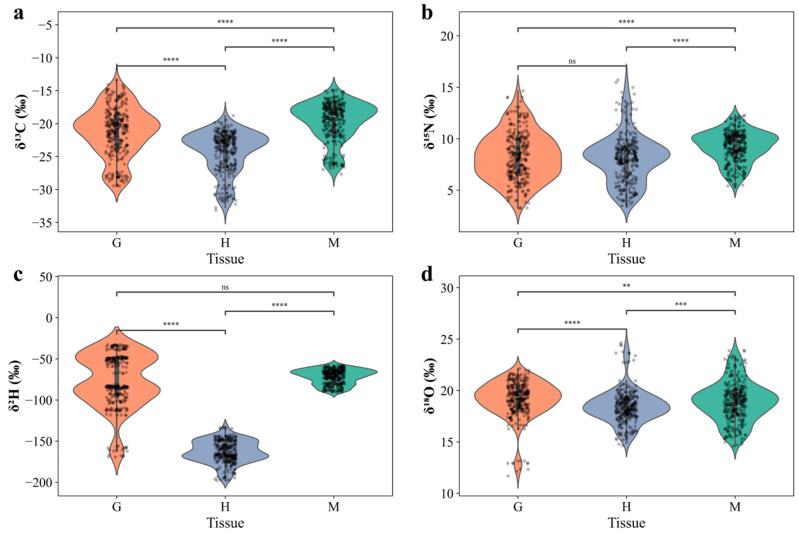
Stable isotope composition of Chinese mitten crabs across different tissues. (**a**–**d**) Violin plots with overlaid data points illustrating the stable isotope values for (**a**) *δ*^13^C, (**b**) *δ*^15^N, (**c**) *δ*^2^H, and (**d**) *δ*^18^O across three tissue types: Muscle (M), Gonad (G), and Hepatopancreas (H). Statistical significance of differences between tissues is indicated by connecting lines and asterisks (** *p* < 0.01, *** *p* < 0.001, **** *p* < 0.0001), while ‘ns’ denotes no significant difference.

Besides the influence of external aquatic environments, significant differences were also observed in the stable isotope composition among different tissues within Chinese mitten crabs. These differences typically reflect the distinct biochemical compositions and metabolic activities of the various tissues. This study analyzed the stable isotope composition of three primary tissue types: muscle, gonad, and hepatopancreas. Statistical results showed that the mean (± standard deviation) *δ*^13^C, *δ*^15^N, *δ*^2^H, and *δ*^18^O values for muscle tissue were −19.65 ± 2.94‰, 9.19 ± 1.53‰, −71.97 ± 9.00‰, and 18.90 ± 1.95‰, respectively. Corresponding values for gonad tissue were −21.18 ± 3.84‰, 8.23 ± 2.40‰, −77.99 ± 33.10‰, and 19.08 ± 1.82‰. Hepatopancreas tissue, however, exhibited markedly different isotopic ratios, with mean *δ*^13^C, *δ*^15^N, *δ*^2^H, and *δ*^18^O values of −24.35 ± 3.18‰, 8.05 ± 2.37‰, −163.27 ± 13.78‰, and 18.42 ± 1.66‰, respectively (Figure 5).

The hepatopancreas exhibited values significantly lower (more negative) than both muscle and gonad tissues. This reflected an extreme depletion of ^2^H in the hydrogen isotopic composition of the hepatopancreas. This phenomenon aligned with the observation for *δ*^13^C, where the hepatopancreas also displayed the lowest *δ*^13^C values. These low values for both isotopes are strongly linked to the hepatopancreas’s primary function as a site for lipid synthesis and storage [48,49]. During biosynthesis, lipids preferentially utilize lighter isotopes (^12^C and ^1^H), leading to a relative depletion of ^13^C and ^2^H in this tissue. Therefore, the high lipid synthesis activity in the hepatopancreas is likely the main physiological mechanism causing its significantly negative *δ*^13^C and *δ*^2^H values [50,51] (DeNiro & Epstein, 1977 and Sessions et al., 1999).

Regarding *δ*^15^N, muscle tissue showed the relatively highest mean value (9.19‰), followed by hepatopancreas and gonad. Muscle tissue, being primarily composed of protein, exhibits a relatively slow protein turnover rate. During nitrogen metabolism, the lighter isotope ^14^N tends to be preferentially lost through excretion, leading to a gradual enrichment of ^15^N in tissues with slower turnover rates like muscle [52,53]. The hepatopancreas, a highly metabolically active organ involved in rapid synthesis and breakdown of various substances, might reflect the nitrogen isotopic signature of recent food intake more directly, or its rapid metabolic turnover could result in a different nitrogen isotopic fractionation pattern compared to muscle [54]. Gonad tissue displayed the lowest *δ*^15^N values, possibly related to selective uptake and utilization of specific amino acids during certain developmental stages or special nitrogen isotopic fractionation effects during the synthesis of reproductive products [55].

Figure 5a illustrates that the *δ*^13^C values of the hepatopancreas were significantly lower than those of muscle and gonad. As the main site for lipid synthesis and storage, the hepatopancreas preferentially utilizes the lighter ^12^C isotope during lipid synthesis, potentially leading to its *δ*^13^C values being more negative compared to muscle and gonad [50,51] (DeNiro & Epstein, 1977 and Sessions et al., 1999). Significant differences were observed in *δ*^18^O values among tissues, but the magnitude of variation was less than that of *δ*^2^H. The hepatopancreas showed slightly lower *δ*^18^O values than muscle and gonad. Tissue *δ*^18^O values are influenced by a combination of factors, including tissue water content, production of metabolic water, and differences in the proportion of oxygen-containing organic compounds (such as proteins, carbohydrates, and lipids) in different tissues and their respective oxygen isotopic compositions [53,54,55].

Overall, relying solely on baseline differences in stable isotope composition to distinguish the geographic origin of Chinese mitten crabs presents a considerable challenge. The isotopic signatures of individuals are shaped by the combined effects of multiple ecological factors, including the isotopic characteristics of the water source, anthropogenic inputs (e.g., pollutants), trophic level within the food web, and specific dietary components. Given this complexity, traditional univariate or simple multivariate statistical methods often lack the capacity to capture the subtle but informative patterns necessary for accurate origin discrimination. In contrast, machine learning approaches offer powerful means of integrating and analyzing such multi-dimensional isotopic data. These methods can identify complex, non-linear patterns and discriminative features among samples from different origins, while accounting for the simultaneous influence of multiple interacting factors. Importantly, as discussed earlier, the significant and biologically interpretable differences in stable isotope composition across different tissues provide additional, high-resolution features for model training. Tissue-specific isotopic fractionation enables the model to detect localized physiological responses to environmental signals, thereby improving the precision and robustness of origin traceability. Based on these considerations, the following section explores the application of machine learning techniques for classifying Chinese mitten crabs according to their geographic origin.

### 3.4. Classification Models

#### 3.4.1. Model Selection

**Figure 6:**
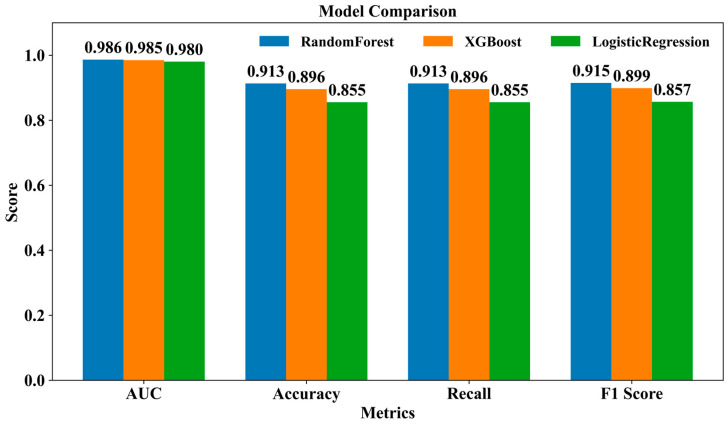
Model performance comparison. Bar chart comparing the performance scores of the Random Forest, XGBoost, and Logistic Regression models across four evaluation metrics—AUC, Accuracy, Recall, and F1 Score—on the test set.

In this study, to achieve accurate classification of Chinese mitten crab samples from different origins, we constructed and evaluated the performance of three machine learning models: Random Forest (RF), XGBoost, and Logistic Regression (LR). The learning curves for all three models, presented in Supplementary Material Figure S1, demonstrated good convergence between training and cross-validation scores with increasing training examples, indicating effective learning by the constructed models and no significant overfitting. The optimal hyperparameters determined for these models are summarized in Supplementary Material Table S2. We compared the final classification performance of the three models using multiple key evaluation metrics, including Area Under the Curve (AUC), Accuracy, Recall, and F1 Score, with detailed results presented in Figure 6 and Table S2. Evaluation results revealed that the RF model exhibited the most robust performance across all four evaluated metrics (Figure 6, Table S2). Notably, the classification accuracy of all our constructed models exceeded the 81.3% accuracy reported in previous related research by Xu et al. [17]. This strongly demonstrated that the RF model possessed excellent capability in this Chinese mitten crab origin classification task, effectively distinguishing samples from different origins and reliably capturing the feature patterns of each class. The XGBoost model also performed well, ranking second; its performance metrics were only marginally lower than those of RF (Figure 6, Table S2). The difference in accuracy between RF and XGBoost was less than 2%, which may not represent a substantial practical advantage in all real-world scenarios. In contrast, the LR model, despite achieving a high AUC, showed considerably lower values for Accuracy, Recall, and F1 Score compared to the other two models (Figure 6, Table S2). This reflects its relatively weaker adaptability in handling data with potential complex non-linear relationships and multi-factor interactions inherent in Chinese mitten crab origin traceability. Considering the overall leading and robust performance across multiple core metrics, the RF model was determined to be the optimal classification model, possessing high generalization ability and practical application potential in this study.

#### 3.4.2. Classification Performance of the Random Forest Model for Different Origins

**Figure 7:**
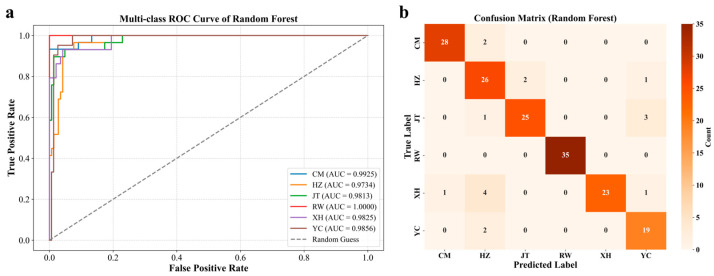
Performance evaluation of the Random Forest model. (**a**) Confusion matrix illustrating the classification results of the Random Forest model, showing the number of samples from each true origin class that were predicted for each origin class. (**b**) Multi-class Receiver Operating Characteristic (ROC) curves for each origin class, plotting the True Positive Rate (TPR) against the False Positive Rate (FPR). The Area Under the Curve (AUC) for each class—CM (AUC = 0.9925), HZ (AUC = 0.9734), JT (AUC = 0.9813), RW (AUC = 1.0000), XH (AUC = 0.9825), and YC (AUC = 0.9856)—is indicated in the legend.

To assess the actual discriminatory capability of the Random Forest model for Chinese mitten crab samples from different origins, we analyzed the model’s performance on the test set, focusing primarily on the Receiver Operating Characteristic (ROC) curve and its Area Under the Curve (AUC) for each class, as well as the model’s confusion matrix (Figure 7). Evaluation results indicated that the model demonstrated excellent overall origin discrimination performance, with AUC values for all origin classes exceeding 0.97 (Figure 7a), significantly surpassing random chance (AUC = 0.5).

Specifically, the model exhibited particularly strong discriminatory power for Chinese mitten crab samples from the RW, CM, XH, and YC, with corresponding AUC values as high as 1.0000, 0.9925, 0.9825, and 0.9856, respectively. It is particularly noteworthy that the model achieved perfect distinction for RW samples (AUC = 1.0000). This strongly confirmed that the combination of sex- and tissue-specific stable isotope features significantly enhanced the model’s identification capability, enabling it to effectively capture the unique imprints of different aquatic environments and potential dietary differences in the isotopic composition of the crabs, thereby achieving high-accuracy origin determination. The confusion matrix (Figure 7b) further provided a detailed validation of the model’s classification performance. The model successfully predicted all samples correctly, especially samples from the RW, highlighting the decisive role of the unique environmental characteristics of the Yangtze River in the model’s discrimination.

It was observed that some mutual misclassifications occurred between samples from YC and HZ (two samples from YC were misclassified as HZ, and one sample from HZ was misclassified as YC). This might be related to the geographical proximity of Yangcheng Lake and Huzhou to the Taihu Lake system, meaning they potentially share some water body environment or food resource characteristics (Figure 1). However, despite these minor misclassifications, the model accurately predicted the vast majority of samples. Overall, these detailed evaluation results, based on ROC curves and the confusion matrix, fully demonstrated the effectiveness and reliability of the Random Forest model for Chinese mitten crab origin traceability, performing particularly well in distinguishing key origins with distinct environmental features, such as the RW, CM, and YC, thus laying a solid foundation for practical application.

#### 3.4.3. Feature Interpretation of the Random Forest Model

**Figure 8:**
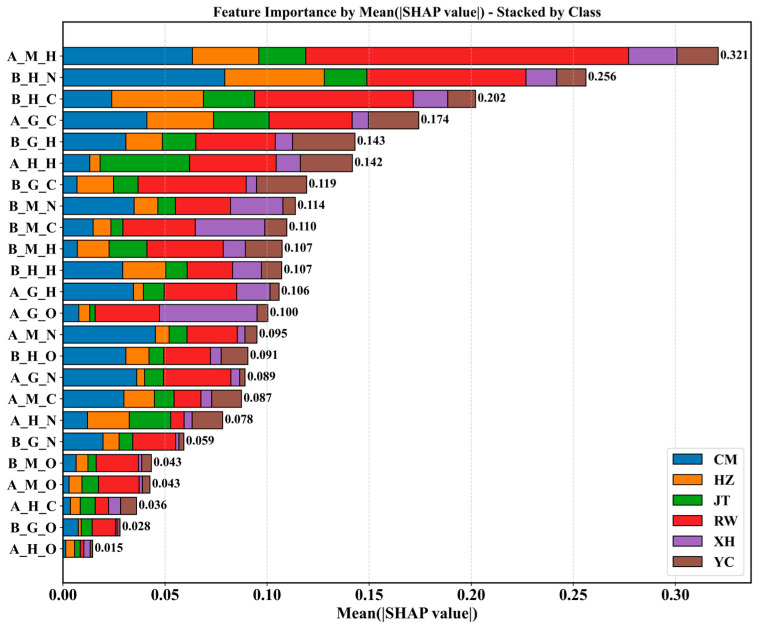
SHAP interpretation of the Random Forest model for Chinese mitten crab origin classification. Global feature importance, represented by the mean absolute SHAP value for each feature averaged across all samples, stacked by the contribution to each origin class. Features are sorted in descending order based on their total mean absolute SHAP value. The numbers next to the bars indicate the total mean absolute SHAP value for each feature.

We constructed an RF model using sex- and tissue-specific stable isotope features of Chinese mitten crabs to achieve accurate origin discrimination. To gain deeper insight into the internal mechanisms behind the model’s predictive decisions and their ecological underpinnings, we applied the SHAP method for interpretation of the optimal model. SHAP values quantitatively represent the contribution of each feature to individual predictions relative to a baseline. By aggregating SHAP values across samples, the method reveals both global feature importance and the relationships between feature values and predicted probabilities [56,57]. Figure 8 summarizes the contribution distributions of 24 features in classifying samples from six origins (JT, CM, XH, HZ, RW, and YC).

From the global feature importance stacked bar plot (Figure 8), ranked by the mean absolute SHAP value across all classes and samples, the *δ*^2^H value of male muscle (A_M_H) exhibited the most significant overall contribution to origin classification, with a mean absolute SHAP value of 0.321. This reflects the substantial contribution of the water body’s *δ*^2^H characteristic in distinguishing crabs from different origins and its most effective accumulation or representation in male muscle tissue. Given the relatively stable turnover rate of muscle tissue, its *δ*^2^H value likely represents the average isotopic signature of the water body the animal inhabited over a longer period. Following in importance were the *δ*^15^N value of female hepatopancreas (B_H_N, 0.256) and the *δ*^13^C value of female hepatopancreas (B_H_C, 0.202). *δ*^15^N and *δ*^13^C are primarily related to diet and food web trophic level. As a metabolically active organ and a major site for lipid synthesis and storage, the hepatopancreas is capable of recording carbon and nitrogen isotopic signals from food [54,58,59]. These features’ high importance suggests that differences in food source composition and trophic structure across origins are key driving factors for discriminating Chinese mitten crabs. The contributions of these globally important features were not confined to a single origin but collectively drove the model’s overall classification capability across multiple categories, with their contribution proportions clearly visualized in the stacked bar plot.

Further analysis of the SHAP summary plots for each origin class (Figure S2a–f) provides detailed insights into how features impact the prediction of specific classes. For the prediction of CM, the *δ*^15^N value of male gonad (A_G_N) showed a prominent contribution; its high values (red points) were strongly associated with significantly positive SHAP values. This indicates that a higher A_G_N value is a key characteristic for identifying samples from the Chongming origin, potentially reflecting a specific trophic pattern or nitrogen accumulation in the male gonad in this area. For origins like HZ and YC, multiple *δ*^2^H-related features (e.M.H, B_M_H, A_H_H) demonstrated important positive contributions, with high values corresponding to positive SHAP. This aligns with the biological basis of *δ*^2^H primarily reflecting water source characteristics, indicating that the isotopic composition of water at these origins exhibits distinctive signatures. Particularly for the discrimination of samples from the RW (Figure S2d), the *δ*^2^H values of male muscle (A_M_H) and female muscle (B_M_H) were among the largest contributors, with their higher feature values (red points) corresponding to positive SHAP values. This significant pattern further supports the model’s strong reliance on *δ*^2^H, which reflects water body differences, to distinguish RW samples. As an open large river system, the RW’s *δ*^2^H characteristic differs significantly from inland farming environments, and this difference is recorded in the relatively stable muscle tissue. Furthermore, it is notable that the *δ*^15^N value of male gonad (A_G_N), which showed an important positive contribution in the prediction of CM, corresponded to negative SHAP values when predicting RW samples (high values → negative SHAP), demonstrating that the same feature can have different and even opposing effects on predicting different origins.

When predicting XH samples (Figure S2e), features showed more complex patterns. For instance, high values (red points) of male gonad *δ*^18^O (A_G_O) tended to produce positive SHAP values, indicating a unique *δ*^18^O characteristic in the water body for the XH origin (reflected in male gonad). However, higher values (red or purple points) of female muscle *δ*^13^C (B_M_C) and female muscle *δ*^15^N (B_M_N) were mainly concentrated in the negative SHAP region. This indicates that, for the XH origin, high *δ*^13^C and *δ*^15^N values in female muscle formed a negative distinction compared to other origins, meaning these values in XH samples might be relatively lower or distributed differently, making high values a basis for the model to exclude XH. Finally, for the prediction of YC (Figure S2f), the *δ*^2^H value of female muscle (B_M_H) was the most prominent contributor, with its high values (red points) strongly associated with positive SHAP values. Concurrently, male muscle *δ*^2^H (A_M_H) and gonad *δ*^13^C (B_G_C, A_G_C) also showed important contributions with high values corresponding to positive SHAP. This again emphasizes the importance of *δ*^2^H reflected in muscle for Yangcheng Lake identification, while *δ*^13^C characteristics in gonad also provided key discriminative information, possibly related to Yangcheng Lake’s unique water quality, food chain structure, or metabolic features during gonad development.

These SHAP-based interpretation results reveal that the RF model effectively captures and utilizes environmental differences for precise classification by integrating stable isotope features reflecting water source (*δ*^2^H, *δ*^18^O) and diet/trophic level (*δ*^13^C and *δ*^15^N) information, combined with their specific expression in different sexes and tissues. Particularly, SHAP analysis intuitively demonstrates how feature value levels influence the direction and strength of prediction probability and highlights differences in contribution across origins, including negative associations where high values decrease prediction probability. This detailed, interpretable analysis significantly enhances our understanding of the model’s decision-making process and ecologically clarifies the discriminative mechanisms of stable isotope fingerprints in Chinese mitten crab origin traceability.

## 4. Conclusions

This study successfully constructed a high-precision origin traceability model for Chinese mitten crabs, leveraging sex- and tissue-specific differences in their stable isotope compositions (*δ*^13^C, *δ*^15^N, *δ*^2^H, and *δ*^18^O) as key features. Among the compared models, the RF model demonstrated superior overall performance across multiple metrics, including AUC, accuracy, recall, and F1 score. It accurately distinguished crab samples from six distinct geographical origins and aquatic environments.

To gain deeper insight into the optimal RF model’s decision-making and ecological relevance, SHAP were applied. SHAP analysis quantitatively assessed feature contributions, clarifying the model’s discriminative basis, and enhancing interpretability and reliability. Globally, male muscle *δ*^2^H (A_M_H), female hepatopancreas *δ*^15^N (B_H_N), and female hepatopancreas *δ*^13^C (B_H_C) were identified as the most influential features. These results underscore the key role of stable isotopes reflecting water source (*δ*^2^H and *δ*^18^O) and diet/trophic level (*δ*^15^N and *δ*^13^C) in differentiating origins.

Importantly, the findings demonstrate that different tissues integrate environmental signals in distinct ways, and that the model effectively exploits these differences for refined classification. By integrating explainable machine learning techniques, this study significantly enhances the accuracy, credibility, and transparency of origin traceability for Chinese mitten crabs. It provides a robust technical framework and theoretical foundation for establishing a reliable, interpretable, and ecologically meaningful traceability system for aquatic products. To further bolster the year-round applicability of this framework, a limitation of this study is its focus on crabs sampled during their final commercial stage (November), which may not fully capture the seasonal variability of environmental isotopes, particularly *δ*^2^H and *δ*^18^O. Future research should therefore expand the temporal sampling window to enhance the model’s robustness for distinguishing crab origins across different seasons and hydrological conditions. Ultimately, this work has important implications for combating origin fraud, protecting consumer rights, and ensuring food safety.

## Data Availability

The original contributions presented in this study are included in the article/Supplementary Materials. Further inquiries can be directed to the corresponding authors.

## Supplementary Materials

The following supporting information can be downloaded at: https://www.mdpi.com/article/10.3390/foods14142458/s1, Table S1: Summary of Statistical Assumption Testing (Normality and Homogeneity of Variance) and Selection of Appropriate Statistical Tests (Kruskal-Wallis) for Isotopic Comparisons by Origin, Environmental Type, and Tissue. Table S2: Summary of classification model parameter configuration and performance indicators. Figure S1: Model learning curves. (a–c) Learning curves illustrating the training score (red) and cross-validation score (green) as a function of the number of training examples for the (a) Random Forest, (b) XGBoost, and (c) Logistic Regression models. The shaded areas represent the standard deviation across cross-validation folds. Figure S2: SHAP summary plots for each geographic origin. (a–f) SHAP summary plots illustrating the impact of stable isotope features on the Random Forest model’s prediction for each of the six origin classes (CM, HZ, JT, RW, XH, and YC). In each plot, a dot represents a sample, colored by its corresponding feature value (blue indicates a low feature value, and red indicates a high feature value).
