# Supplementary material for: Origin Traceability of Chinese Mitten Crab (Eriocheir sinensis) Using Multi-Stable Isotopes and Explainable Machine Learning
Source: Foods. 2025 Jul 13;14(14):2458. doi: 10.3390/foods14142458 (PMC12294520; doi:10.3390/foods14142458)
Supplement: Supplementary file 1 [file foods-14-02458-s001.zip › foods-3693439-supplementary.pdf]

Supplementary Material

Origin Traceability of Chinese Mitten Crab (*Eriocheir sinensis*) Using Multi-Stable Isotopes and Explainable Machine Learning

Danhe Wang [1,2,†], Chunxia Yao [1,2,†], Yangyang Lu [1,2], Di Huang [1,2], Yameng Li [1,2], Xugan Wu [3], Weiguo Song [1,2,*] , Qinxiong Rao [4,*]

[1] The Institute of Agro-food Standards and Testing Technology, Shanghai Academy of Agricultural Sciences, Shanghai, 201403, China; wdh@saas.sh.cn (D.W.); chunxiayao2007@saas.sh.cn (C.Y.); yyanglz@163.com (Y.Lu); hdsaas@126.com (D.H.); liyameng@saas.sh.cn (Y.Li)

[2] Shanghai Service Platform of Agro-products Quality and Safety Evaluation Technology, Shanghai 201403, China

[3] Research Centre on Fish Nutrition and Environmental Ecology of Ministry of Agriculture and Rural Affairs, Shanghai Ocean University, Shanghai 201306, China; xgwu@shou.edu.cn (X.W.)

[4] Shanghai Co-Elite Agri-food Testing Technical Service Co., Ltd, Shanghai 201403, China

* Correspondence: songweiguo@saas.sh.cn (W.S.); qinxiongrao@saas.sh.cn (Q.R.); Tel.: +86-21622-02796

† These authors contributed equally to this work

.

Table S1. Summary of Statistical Assumption Testing (Normality and Homogeneity of Variance) and Selection of Appropriate Statistical Tests (Kruskal-Wallis) for Isotopic Comparisons by Origin, Environmental Type, and Tissue.

Analysis Category	Feature	Group	Shapiro Wilk p	Normality Pass	Levene p	Homogeneity of Variance	Test Method
By Origin	$\delta^{12}C$	CM	0.00E+00	No	0.00E+00	No	Kruskal-Wallis test
		HZ	7.70E-03	No			
		JT	0.00E+00	No			
		RW	0.00E+00	No			
		XH	2.00E-04	No			
		YC	6.10E-03	No			
	$\delta^{15}N$	CM	7.70E-03	No	0.00E+00	No	Kruskal-Wallis test
		HZ	7.80E-03	No			
		JT	2.44E-01	Yes			
		RW	1.00E-04	No			
		XH	3.92E-02	No			
		YC	1.85E-01	Yes			
	$\delta^{2}H$	CM	0.00E+00	No	6.80E-03	No	Kruskal-Wallis test
		HZ	0.00E+00	No			
		JT	0.00E+00	No			
		RW	0.00E+00	No			
		XH	0.00E+00	No			
		YC	0.00E+00	No			
	$\delta^{18}O$	CM	0.00E+00	No	1.98E-02	No	Kruskal-Wallis test
		HZ	0.00E+00	No			
		JT	8.90E-03	No			
		RW	1.00E-04	No			
		XH	4.47E-02	No			
		YC	0.00E+00	No			
By Env. Type	$\delta^{12}C$	Pond	0.00E+00	No	0.00E+00	No	Kruskal-Wallis test
		Lake	6.10E-03	No			
		River	0.00E+00	No			
	$\delta^{15}N$	Pond	0.00E+00	No	0.00E+00	No	Kruskal-Wallis test
		Lake	1.85E-01	Yes			
		River	1.00E-04	No			
	$\delta^{2}H$	Pond	0.00E+00	No	5.92E-02	Yes	Kruskal-Wallis test
		Lake	0.00E+00	No			
		River	0.00E+00	No			
	$\delta^{18}O$	Pond	1.70E-03	No	5.57E-02	Yes	Kruskal-Wallis test
		Lake	0.00E+00	No			

By Tissue	$\delta^{12}C$	River	1.00E-04	No	5.00E-04	No	Kruskal-Wallis test
		G	0.00E+00	No			
		H	0.00E+00	No			
		M	0.00E+00	No			
	$\delta^{15}N$	G	4.03E-02	No	0.00E+00	No	Kruskal-Wallis test
		H	0.00E+00	No			
		M	0.00E+00	No			
	$\delta^{2}H$	G	0.00E+00	No	0.00E+00	No	Kruskal-Wallis test
		H	4.00E-04	No			
		M	0.00E+00	No			
	$\delta^{18}O$	G	0.00E+00	No	5.40E-03	No	Kruskal-Wallis test
		H	0.00E+00	No			
		M	5.10E-03	No			

Table S2. Summary of classification model parameter configuration and performance indicators.

Models	Data Preprocessing	Hyperparameters	Accuracy	Recall	F1 Score	AUC
Random Forest (RF)	Missing Value Imputation<br>Categorical Encoding	Number of Estimators = 70<br>Maximum Depth = 20<br>Minimum Samples Split = 5<br>Minimum Samples Leaf = 2<br>Splitting Criterion = entropy<br>K-Fold = 5 (Stratified)	0.913	0.913	0.915	0.986
XGBoost	Missing Value Imputation<br>Categorical Encoding	Number of Estimators = 100<br>Maximum Depth = 7<br>Fraction of Features per Tree = 0.7<br>Fraction of Samples per Tree = 0.7<br>Minimum Samples Leaf = 2<br>Learning Rate = 0.1<br>K-Fold = 5 (Stratified)	0.896	0.896	0.899	0.985
Logistic Regression (LG)	Standardization<br>Categorical Encoding	Regularization Strength (C) = 10<br>Penalty = L2<br>Optimization Algorithm = lbfgs<br>K-Fold = 5 (Stratified)	0.855	0.855	0.857	0.980

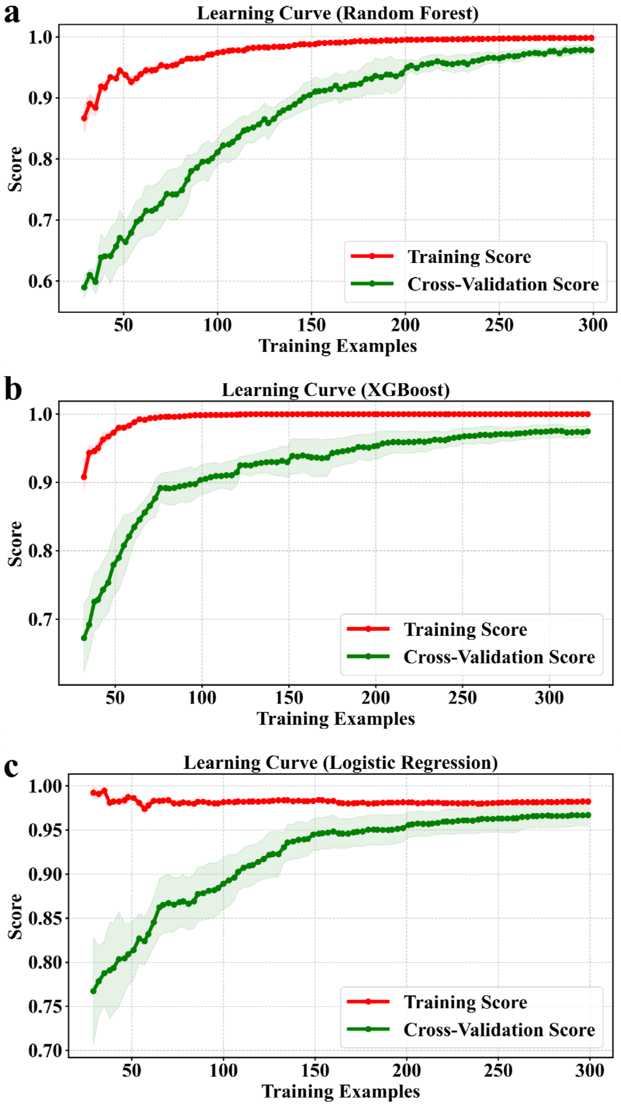

Figure S1. Model learning curves. (a-c) Learning curves illustrating the training score (red) and cross-validation score (green) as a function of the number of training examples for the (a) Random Forest, (b) XGBoost, and (c) Logistic Regression models. The shaded areas represent the standard deviation across cross-validation folds.

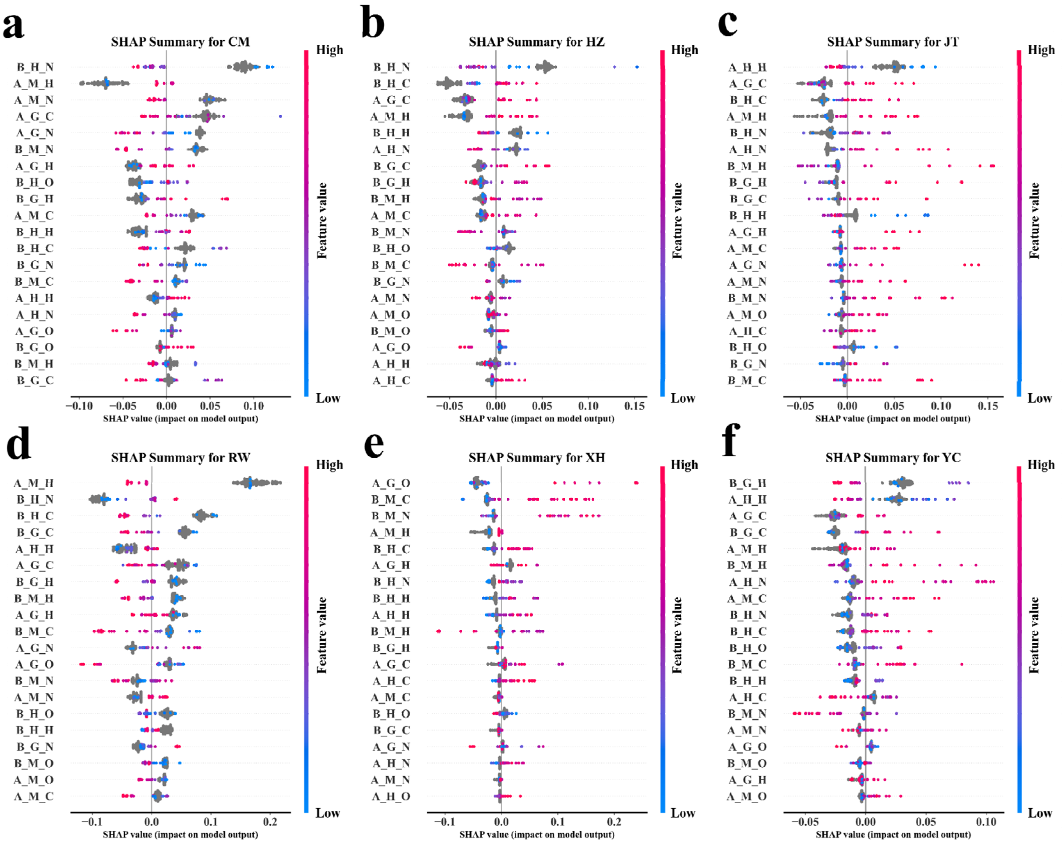

Figure S2. SHAP summary plots for each geographic origin. (a–f) SHAP summary plots illus-trating the impact of stable isotope features on the Random Forest model's prediction for each of the six origin classes (CM, HZ, JT, RW, XH, YC). In each plot, a dot represents a sample, colored by its corresponding feature value (blue indicates a low feature value, and red indicates a high feature value).
